# Training Courses in Laryngeal Nerve Monitoring in Thyroid and Parathyroid Surgery- The INMSG Consensus Statement

**DOI:** 10.3389/fendo.2021.705346

**Published:** 2021-06-18

**Authors:** Che-Wei Wu, Gregory W. Randolph, Marcin Barczyński, Rick Schneider, Feng-Yu Chiang, Tzu-Yen Huang, Amanda Silver Karcioglu, Aleksander Konturek, Francesco Frattini, Frank Weber, Cheng-Hsin Liu, Henning Dralle, Gianlorenzo Dionigi

**Affiliations:** ^1^ International Thyroid Surgery Center, Department of Otolaryngology-Head and Neck Surgery, Kaohsiung Medical University Hospital, Kaohsiung Medical University, Kaohsiung, Taiwan; ^2^ Center for Liquid Biopsy and Cohort Research, and Faculty of Medicine, College of Medicine, Kaohsiung Medical University, Kaohsiung, Taiwan; ^3^ Department of Otolaryngology-Head and Neck Surgery, Kaohsiung Municipal Siaogang Hospital, Kaohsiung Medical University, Kaohsiung, Taiwan; ^4^ Department of Otolaryngology, Massachusetts Eye and Ear Infirmary, Boston, MA, United States; ^5^ Department of Surgery, Massachusetts General Hospital, Harvard Medical School, Boston, MA, United States; ^6^ Department of Endocrine Surgery, Third Chair of General Surgery, Jagiellonian University Medical College, Krakow, Poland; ^7^ Department of Surgery, University Hospital Halle, Martin-Luther-University, Halle-Wittenberg, Germany; ^8^ Department of Otolaryngology, E-Da Hospital, School of Medicine, College of Medicine, I-Shou University, Kaohsiung, Taiwan; ^9^ Division of Otolaryngology-Head and Neck Surgery, Department of Surgery, NorthShore University HealthSystem, Evanston, IL, United States; ^10^ Department of Surgery, Ospedale di Circolo, ASST Settelaghi, Varese, Italy; ^11^ Department of General, Visceral and Transplantation Surgery, University of Duisburg-Essen, Essen, Germany; ^12^ Department of Human Pathology in Adulthood and Childhood “G. Barresi”, University Hospital G. Martino, University of Messina, Messina, Italy

**Keywords:** intraoperative neural monitoring, vocal cord paralysis, vagus nerve, recurrent laryngeal nerve, external branch of superior laryngeal nerve, training courses, parathyroid surgery, thyroid surgery

## Abstract

Intraoperative neural monitoring (IONM) is now an integral aspect of thyroid surgery in many centers. Interest in IONM and the number of institutions that perform monitored thyroidectomies have increased throughout the world in recent years. For surgeons considering the introduction of IONM in their practice, specific training in IONM devices and procedures can substantially shorten the learning curve. The International Neural Monitoring Study Group (INMSG) has been at the forefront of IONM technology and procedural adoption since the introduction of neural monitoring in thyroid and parathyroid surgery. The purpose of this document is to define the INMSG consensus on essential elements of IONM training courses. Specifically, this document describes the minimum training required for teaching practical application of IONM and consensus views on key issues that must be addressed for the safe and reliable introduction of IONM in surgical practice. The intent of this publication is to provide societies, course directors, teaching institutions, and national organizations with a practical reference for developing IONM training programs. With these guidelines, IONM will be implemented optimally, to the ultimate benefit of the thyroid and parathyroid surgical patients.

## Introduction

Intraoperative neural monitoring (IONM) is now an integral aspect of thyroid surgery in many centers. Interest in IONM, the number of institutions that perform, and societies that recommended monitored thyroidectomies have increased throughout the world in recent years (1–16). A recent international survey (16) of over 1,000 surgeons revealed that IONM is highly prevalent with 83% of surgeons using IONM in some or all of their cases. Reasons for use included patient request, preoperative vocal cord palsy, thyroid cancer, substernal goiter and most notably for reoperative surgery where the rate of use was 95%. The body of knowledge in IONM has also rapidly evolved in recent years with the introduction of new, less invasive monitoring devices, as well as the publication of prospective randomized clinical trials, multi-center studies, cost analyses, studies of ethical/medicolegal issues, clinical guidelines, and standards for the recurrent laryngeal nerve (RLN) and external branch of the superior laryngeal nerve (EBSLN) monitoring (1–4, 11, 17–34). Additionally, current technology enables the use of continuous-IONM (C-IONM) to analyze nerve monitoring signal loss and recovery under periodic vagus nerve (VN) stimulation and to understand the relationship between signal loss and early postoperative vocal fold palsy (5, 35–38). Up-to-date structured courses are needed to introduce the clinical, legal and research implications of these developments, in order that monitoring be done at the highest optimal and most current standards.

**Figure 1:**
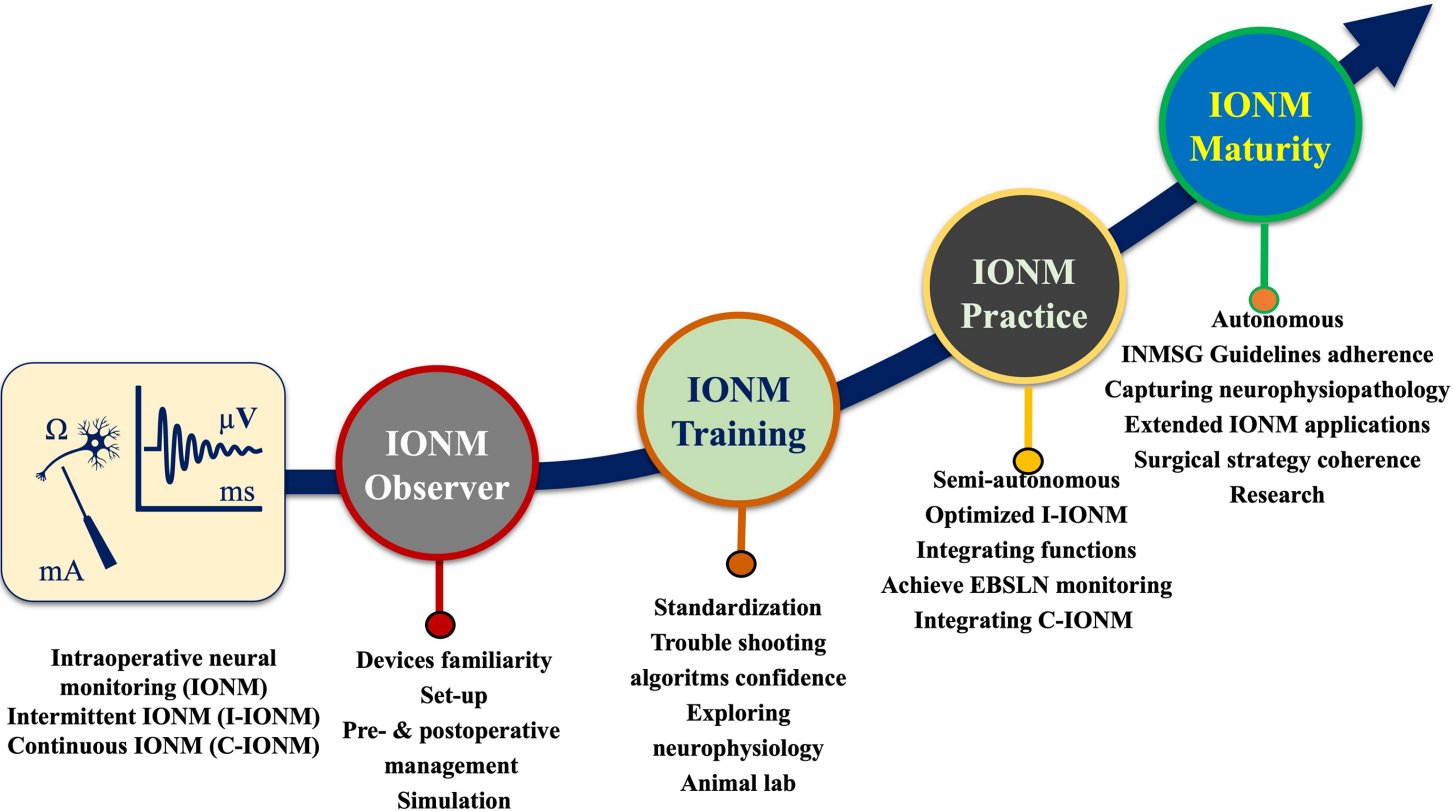
A journey to autonomous IONM operations. Training evolution for implementation of basic and advanced IONM system (IONM needs, stages and benefits).

As IONM use in thyroid/parathyroid surgery is increasing, surgical residency programs have begun including IONM courses in their core curricula. Competency in the use of IONM technology in thyroid/parathyroid surgery is being tested similar to the use of other technologies (e.g., laparoscopic technology) in other specialties such as neurosurgical spine surgery (39), the training evolution for implementation of basic and advanced IONM system (IONM needs, stages and benefits) will improve the surgical residency and surgeon’s practice and maturity of autonomous IONM operations (**Figure 1**).

Despite the growing need for and acceptance of IONM training, there remains a lack of professional certification by national or international thyroid societies (40). Currently, surgeons primarily receive IONM training through industry-sponsored courses that provide limited opportunities for certification and may be biased. Additionally, these non-standardized courses in IONM have not been vetted by professional groups and may not provide the optimal clinical education based on the most recent scientific literature.

The International Nerve Monitoring Study Group [INMSG(www.inmsg.org)] has been at the forefront of IONM technology and procedural adoption since the introduction of neural monitoring in thyroid/parathyroid surgery (1). Specifically, the INMSG has had leading roles in the development and evolution of international guidelines for laryngeal nerve monitoring (1–4). The INMSG has also addressed key issues in IONM training and specialization. The INMSG Board which is global and multidisciplinary in composition, has developed national and international training courses for general surgery and surgical specialties, including endocrine, head and neck, and Otolaryngologic or ENT surgery, in Europe, the US and Asia (**Supplementary File 1**). National IONM Study Groups have also developed IONM courses in collaboration with INMSG. The aims of training courses are (i) to address the increased interest in IONM and (ii) to discuss recent research in IONM as well as guidelines and standards for IONM practice. Since better clinical outcomes of IONM are associated with well-coordinated introduction to and training in new IONM technologies and procedures, consensus statements are needed to ensure an orderly process of incorporating these technologies in surgical practice (4, 41, 42). For surgeons considering the introduction of IONM in their practice, specific training in IONM devices and procedures can substantially shorten the learning curve (43). Notably, surgeons who adopt IONM tend to modify their surgical practices and dissection techniques and may have improved outcomes (43, 44).

The purpose of this document is to define the INMSG consensus on essential elements of IONM training courses. Specifically, this document describes the minimum training required for teaching practical application of IONM and consensus views on key issues that must be addressed for safe and reliable introduction of IONM in surgical practice. With these guidelines we feel IONM will be implemented optimally, to the ultimate benefit of thyroid/parathyroid surgical patients.

## Consensus Statement and Implications

### Training Course Requirements

#### Course Leadership Experience Level

##### General Requirements

Course leaders must have robust experience and have a national board qualification in general, endocrine, or otolaryngology-head and neck surgery (45, 46). By acting as a chair, the organizer of the IONM course should be able to provide expertise based on relevant experience and qualifications. A minimum of 50 cases per year, for no less than three years, should provide the course chair with sufficient experience for their role as the course director. Since the focus of the IONM Course is applying and managing IONM technology in thyroid/parathyroid surgery, course faculty and instructors require profound knowledge of endocrine neck surgery to provide the optimal environment for learning comprehensive implementation and management of IONM. The course should also emphasize patient safety and should ideally be enjoyable for participants.

##### IONM Requirements

###### Scope of Basic Course

**Figure 2:**
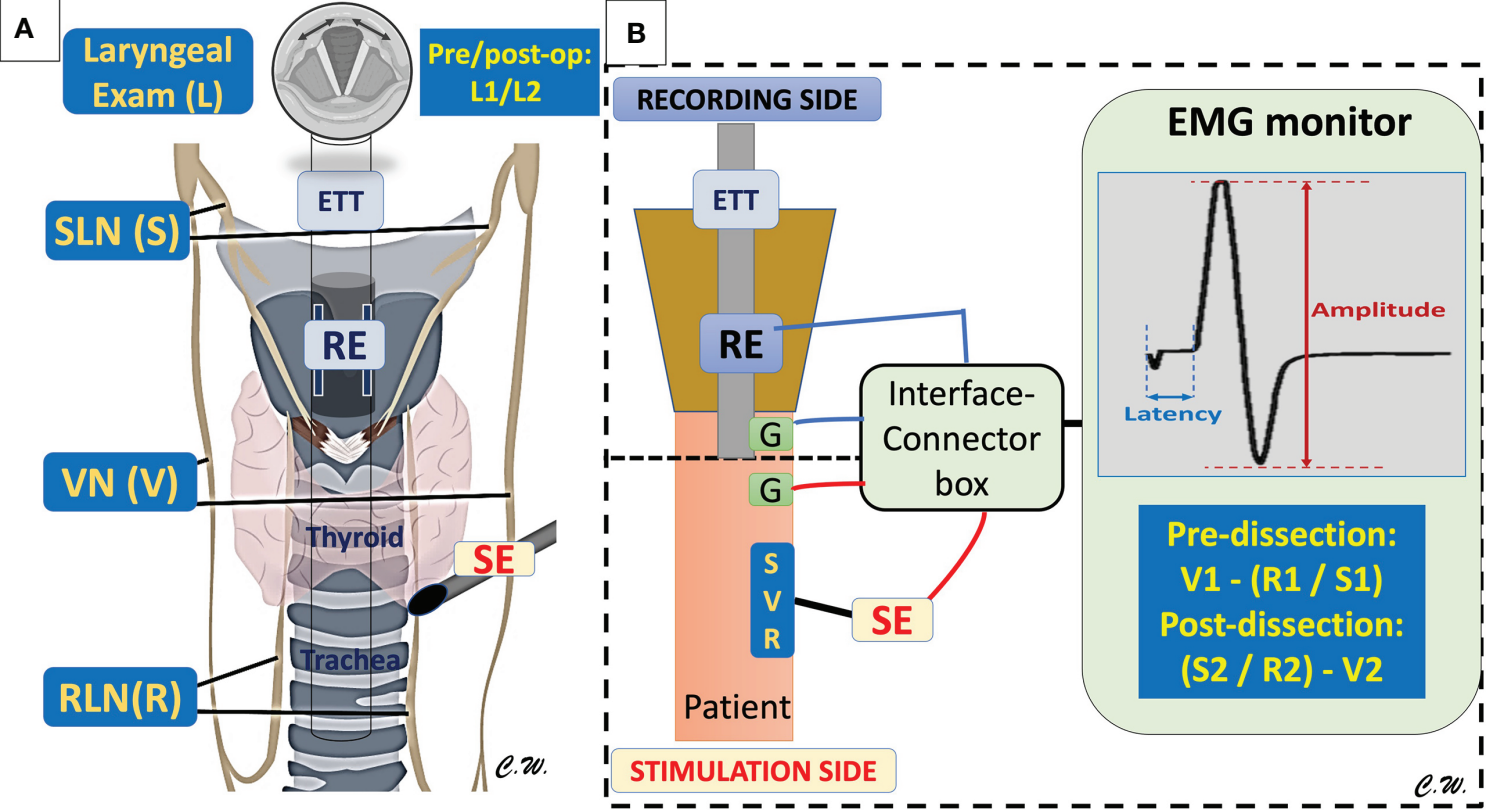
The Basic laryngeal nerve anatomy/electrophysiology and standard monitoring equipment setup/procedures. **(A)** The basic equipment included the recording electrodes (RE) connected to the endotracheal tube (ETT) to place in contact with the bilateral vocal fold, the neural stimulating electrodes (SE) to stimulate the external branch of superior laryngeal nerve (SLN)(S), the Vagus nerve (VN)(V), and the recurrent laryngeal nerve (RLN)(R) during thyroid and parathyroid surgery. The standard procedure for performing IONM should include the laryngeal examination (L) before and after surgery(L1-L2), stimulating the SLN(S) before and after upper thyroid pole dissection (S1-S2), and stimulating the VN(V) and RLN(R) before and after the dissection. **(B)** Standard equipment setup (Recording (RE)/Stimulation (SE) side, ground (G) electrodes, interface connector box and monitor connections. The stimulating electrode (SE) can be used for mapping, localization, and identification of the SLN (S), VN (V), and RLN (R), and the evoked laryngeal EMG waveform can be viewed on the EMG monitor screen, and the amplitude and latency changes can be monitored during surgery.

Course faculty and instructors require full knowledge of laryngeal nerve anatomy/electrophysiology (VN, EBSLN, RLN) and profound experience in the standard use of IONM, including laryngeal examination before and after surgery*(L1-L2)*, collaboration with the anesthesiologist (neuromuscular blockage agent selection and EMG endotracheal tube placement), standard equipment setup (Recording/Stimulation side electrodes, interface box and monitor connections), stimulating the EBSLN before and after upper thyroid pole dissection*(S1-S2)*, and stimulating the VN and RLN before and after the dissection*(V1-R1-R2-V2)* (**Figure 2**).

###### Knowledge of Common Pitfalls and Ability to Use Troubleshooting Algorithms

**Figure 3:**
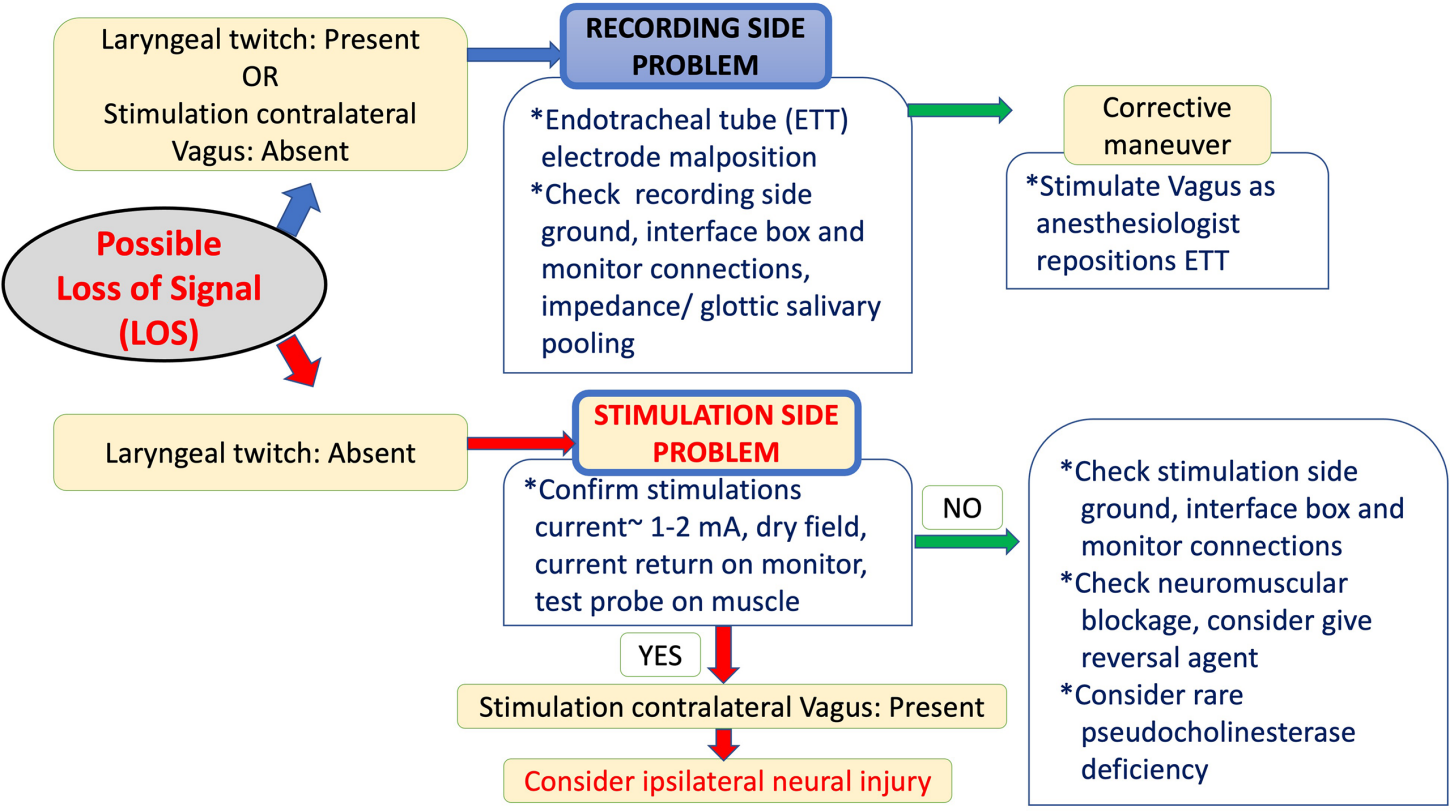
The International Neural Monitoring Study Group (INMSG) recommendation for loss of signal (LOS) evaluation and troubleshooting algorithms.

The course should clarify the limitations of IONM, demonstrate how to identify artifacts by applying algorithms and systematic troubleshooting procedures, foster the development of skills to optimize interaction with the anesthesiologist and manage LOS (**Figure 3**). It is for these reasons that a course leader have a strong foundation and sufficient experience as outlined above, as well as a clear understanding of how these limits impact surgical strategy.

###### Adherence to IONM Guidelines

The INMSG has published guidelines (1–4) for using IONM to monitor the RLN and EBSLN in thyroid/parathyroid surgery. The INMSG has also provided guidelines for managing LOS and avoiding RLN injury. These guidelines should be discussed comprehensively in the course to train and improve the participants’ practice and maturity of autonomous IONM operations (**Figure 1**).

### Anesthesiologist

When performing thyroid/parathyroid surgery, the surgeon shares the responsibility for implementing IONM. IONM training therefore must incorporate discussion of collaboration between the anesthesiologist and the surgeon (47, 48). The course should include an expert anesthesiologist, where possible, to provide training in maintaining optimal intraoperative communication between the anesthesiologist and the surgeon.

### Basic and Advanced Courses

Courses offered by IONM training centers should include basic and advanced Courses.

#### Basic IONM Course

Basic course content should include basic knowledge of IONM, common errors in application, how IONM may assist in RLN identification and RLN mapping in both routine and difficult thyroid/parathyroid operations. Training should include standard IONM procedures, such as acquiring the essential dataset by “*L1-V1-R1-S1-S2-R2-V2-L2”* stimulation/laryngeal examination (**Figure 2**) and LOS evaluation and troubleshooting algorithms (**Figure 3**).

#### Advanced IONM Course

In addition to basic course content (which may be abbreviated depending on the audience), advanced course content should include C-IONM and EBSLN monitoring, quantitative and qualitative normative parameters for evaluating and interpreting EMG of the VN and RLN, and voice outcome analysis.

### Course Setting and Additional Integrated Training Facilities

#### IONM Course Setting

The course should be delivered using modern IONM equipment and monitoring software with both audio and graphic monitoring documentation.

##### Live OR Demonstration

Demonstration of IONM applications in the OR may be done during live surgery or by video of live surgery. While in-person live format limits the number of attendees, it enables discussion of IONM techniques among individual participants in real time. Video format with interactive discussion is suitable for larger groups.

##### IONM Course Content, Implementing and Marketing

Course content should facilitate teaching both theoretical and practical skills in IONM so that participants who have completed the course should understand the fundamental steps and routine procedures (**Table 1**) for performing a safe IONM procedure.

**Table 1:** INMSG Recommendation for Implementing IONM technology.

Familiarization with IONM devices and procedures Training in IONM devices and procedures Hands-on practice using appropriate training models before actual IONM use in patients Assessment of ability to perform IONM safely before actual IONM use in patients Disclosure of IONM use to patient Meticulous recording and monitoring of IONM use in surgery, including procedures (L1-L2 laryngeal examination, and S1-S2/V1-R1-R2-V2 stimulation) and outcomes (rates/types of LOS, mechanisms of nerve injury, and rates vocal cord paresis/paralysis).

INMSG, International Nerve Monitoring Study Group; IONM, Intraoperative neural monitoring; L1-L2, laryngeal examination before and after surgery; S1-S2, stimulating the EBSLN before and after upper thyroid pole dissection; V1-R1-R2-V2, stimulating the VN and RLN before and after the dissection.

Lectures on IONM in thyroid/parathyroid surgery should summarize basic knowledge in RLN anatomy and function, as well as advantages and limitations of IONM. Course learning objectives should be clearly stated to provide structure to the course and to inform participants on the content and level of the course. The optimal course content is listed in **Table 2**.

**Table 2:** INMSG Recommendation for IONM training course content.

RLN-EBSLN-VN anatomy and pathophysiology of injury states Review of literature on IONM Medicolegal and ethical considerations (including consent) Cost considerations Basic electrophysiology Basic endotracheal EMG tube placement Definition of LOS LOS and adjustments to extent of surgery IONM for invaded RLN Basic troubleshooting algorithms EBSLN monitoring C-IONM Review of INMSG guidelines	

INMSG, International Nerve Monitoring Study Group; IONM, Intraoperative neural monitoring; RLN, recurrent laryngeal nerve; EBSLN, external branch of superior laryngeal nerve; VN, vagus nerve; LOS, loss of signal; C-IONM, Continuous Intraoperative neural monitoring.

The Course can be marketed *via* the Internet, the INMSG website/member lists, the World Congress on Thyroid Cancer (WCTC), and individual medical technology companies (under the approval of and collaboration with INMSG).

#### Participants and Training Surgeons

##### Course Size

For adequate teaching efficiency, the number of participants should not exceed a number that can be accommodated optimally in the live OR. We have found that a small group of 8-10 participants enables in-depth discussion of diverse perspectives and enables an intimate experience and exchange of ideas. A small group also facilitates and encourages interaction among all participants and faculty and the development of personal relationships among the participants, which improves educational outcomes and may lead to future long-term professional collaboration.

##### Participants in IONM Training Courses

In recent years course participants have included surgeons with varying experience and backgrounds, including general surgery, otolaryngology-head and neck surgery, endocrine surgery, and thoracic surgery. Anesthesiologists often attend as well. Other potential participants include surgical nursing staff and IONM technicians. Participants with widely varying levels of knowledge, expertise and experience can benefit from the course, including currently practicing clinicians, residents, young surgical trainees, and medical students. The participant is recommended to select the appropriate course level (basic or advanced) however, extensive experience in IONM is not required to obtain a practical benefit from the course.

### Syllabus

Course organizers should determine and make clear the expertise level at which the course is aimed. Less experienced participants may require pre-course training modules (online videos and/or required reading) in order to ensure the minimum working knowledge needed to benefit optimally from the course. The syllabus should contain information about the course program as well as pertinent recent literature on IONM. Literature summary materials may be provided to participants through an online link or on a USB flash drive. Ideally, the syllabus should be provided to participants at least one month before the course begins.

### Surgical Procedures

#### Live Surgical Demonstration and Case Selection

Demonstration of surgical applications of IONM should be limited to thyroid/parathyroid surgery. Cases selected for demonstration in the course should be limited to routinely performed conventional thyroidectomies or routine parathyroidectomies. Patients should be selected carefully and should be limited to those with minor comorbidity to minimize risk of last minute cancellation. If possible, patients should be limited to those who previously follow-up or treated by the surgeon performing the live demonstration. Written informed consent to undergo “live” surgery for educational purposes must be obtained from the patient. A list of reserve patients should be prepared well in advance so that a replacement can be available on short notice.

#### Animal Models

The course leader must comply with the procedures for using experimental animal models at the animal laboratory where the course is delivered (see *Actionable Recommendations*).

### Duration of the IONM Course

The complete course, including lectures, discussions, case studies, and/or demonstrations should ideally be designed for completion in 2 days.

### Financing

Course financing should be responsibility of the institution sponsoring the course and/or an official society. Conflicts of interest of the course faculty and course sponsorship must be declared.

### Feedback Method, Questionnaires, and Conclusion of the IONM Course

The Course should conclude with an interactive question-and-answer session moderated by the course leader. Feedback from participants at the conclusion of the course is important for quality control and further refinement of the course. Participants should complete a course evaluation form to document their assessments of the quality and utility of the Course and to provide feedback for future modifications and improvements in course content and/or structure. Administering a pre-/post-test can provide course directors with insight into the educational benefit of their course and how it can be improved. Participants should also complete the 37-item questionnaire developed by the INMSG for self-assessment of the benefit received from the course ***(*** **Supplementary File 2** ***)***.

### Certificate of Attendance

Participants who successfully complete the post-test and evaluation should receive a certificate of attendance.

## Actionable Recommendations

### Improving Proficiency in IONM Procedures

#### Technology Applicable for IONM Simulation

Surgical simulation is an effective and sustainable adjunct to surgical training (49, 50). Implementation of surgical simulations in various surgical specialties has been discussed extensively, including effective designs, practical implementation issues, and analysis of simulation results (49–51). Use of surgical simulations for educational purposes is feasible in training courses and is enjoyable for trainees (52, 53). One currently available IONM simulation technology is NIM™SAM-T, a teaching and demonstration tool used to illustrate basic and advanced principles of nerve monitoring (54).

#### Online Courses and Outreach

The INMSG offers online training programs in accordance with recent e-learning trends. Online programs provide the capability to reach participants in widely dispersed geographic locations (without limitation to time and cost of travel), to deliver training courses under special conditions (e.g., the Covid19pandemic), to follow-up with participants, and to share IONM materials quickly and easily in various formats (e.g., videos, photographs, word/pdf processing files, presentation files, etc.). To provide IONM experts with updated knowledge of new developments, the INMSG is in the process of increasing its interaction with IONM experts through use of various communication formats and technologies, including webinars, chat applications, and internet forums/message boards.

#### IONM Training Courses in Animal Laboratories

In the past 20 years, INMSG has also implemented training courses in animal laboratories. Animal studies are an essential component of IONM research and are a crucial educational tool for disseminating basic IONM knowledge. Animal research has been instrumental in development of IONM standards and in the enormous advances in IONM quality in recent years (55).

##### Animal Models

Currently, most training programs and research use medium-sized animals for experimental models, most typically canine(dog) models (56–59) and porcine(swine/mini-pig) models (60–77). Dog models of laryngeal function and the RLN are well-established and closely mimic the anatomy, size, and physiology in humans. The porcine model was the earliest animal model used in RLN research. Its medium size enables easy handling, and experimental animals are widely available and relatively inexpensive.

##### Animal Course Format

###### Duration, Timing, and Preparation

The duration may be one half-or full day, optimally after the standard 1-to 2-day course of lectures with live surgical demonstration. Before the animal course, the instructors should briefly discuss the neck anatomy of the experimental model and the details of the procedures.

###### Animal Course Set Up and Purpose

Live animal surgery should include veterinarians and anesthesiologists for technical assistance in the animal preparation and anesthesia. Additionally, the protocol should be reviewed and approved in advance by the animal care and use committee of the host institution to ensure that the course complies with national/international regulations and with the 3Rs (“replacement, reduction and refinement”) principle of animal experiments (73). The main advantage of animal modeling is that, unlike human surgery, EMG correlates of RLN injury can be studied quantitatively. Therefore, for the IONM trainee, animals are useful for demonstrating the relationship between neural injury and EMG decrements (55, 64).

##### Animal Course Protocol

###### Animal Preparation/Anesthesia

During anesthesia induction and tracheal intubation, the instructors should discuss the effects of anesthetics and the principles of EMG tube placement and fixation (48, 78, 79).

###### Nerve Monitoring Setting

The instructors should introduce the key features of the stimulation/recording equipment, grounding electrodes, and associated connections at the interface-connector box and monitor. By the end of the course, participants should be familiar with electrode connections and stimuli/threshold settings for the monitoring system.

###### Nerve Preparation

Participants should learn basic IONM applications by using an I-IONM stimulation probe for mapping and localization before visual identification of target nerves, including EBSLN, RLN, and VN (34). Nerves can then be identified and exposed for the experiments.

###### Basic Electrophysiology Study

***a. Baseline EMG Responses.*** Participants can learn normative EMG responses (different waveform/latencies, minimal/maximal stimulus levels, and stimulus-response relationships) by testing EMG responses in the EBSLN-RLN-VN (34, 69).

***b. Stimulating Electrodes.*** If different I-IONM or C-IONM stimulation electrode types are available (e.g., monopolar probes, bipolar probes, stimulation probes/dissectors, and APS™ electrode), sensitivity can be tested and compared in different stimulation probes/dissectors used at different distances and in different fascia (69, 80). The procedures for APS™ vagal electrode placement and monitoring can be practiced repeatedly to enable participants to master clinical skills (73, 77).

***c. Recording Electrodes.*** Participants can compare the effects of mispositioned or displaced EMG tubes that leads to false LOS due to rotation or upward/downward EMG tube displacement). Participants can also learn troubleshooting algorithms and corrective maneuvers for mispositioned EMG tubes (67).

###### Advanced Nerve Injury Study

After completion of repeatable electrophysiology studies, participants can perform nerve injury experiments by testing the nerve segments from proximal to distal segments (e.g., caudal to cranial part of the RLN). A typical use of C-IONM is to confirm and compare patterns of change in real-time evoked laryngeal EMG signals during and after RLN injuries caused by different mechanisms [e.g., traction injuries (58, 59, 61, 64, 65) versus thermal injuries (62–64, 68, 74, 75)](**Table 3**). The I-IONM probe can be used to confirm and map the injury area and to compare proximal and distal EMG.

## Discussion

Over the past decade, the use of IONM in thyroid and parathyroid surgery has become well established and is increasing accepted across the world. Recent data from UKRETS (13), SQRTPA (14), EUROCRINE (15) and international surveys (16) give incite into the current practices in IONM. These data show that IONM is performed by a large majority of thyroid surgeons and helps identify both the RLN anatomically and recognize injury in a higher percentage of cases than without IONM. In addition, IONM reduces the risk of RLN palsy, both temporary and permanent injury.

As IONM use in thyroid/parathyroid surgery is increasing, surgical residency programs have begun including IONM courses in their core curricula. An IONM training course aims to increase basic knowledge in IONM, including common errors in IONM application, and to increase competence in nerve identification, mapping, and functional preservation, not only in the routine management of the RLN and the EBSLN, but also in complex thyroid/parathyroid surgery. By the end of the course, participants should have practical knowledge in standard use of IONM, including positioning the endotracheal tube, interacting with the anesthesiologist, differentiating between electromyography(EMG) signals and artifacts, and applying troubleshooting algorithms. Additionally, participants should know the centrally important procedures for differentiating the key elements of technical/equipment failure in IONM from true loss of signal (LOS) during thyroid/parathyroid surgery (**Figures 2**, **3**). This is a core IONM functionality and will guide the participants in future IONM practice and maturity (**Figure 1**).

The INMSG is the leading professional association in neurophysiological assessment and monitoring of laryngeal nerves in thyroid and parathyroid surgery (1 ***–*** 4). The group is comprised of a multidisciplinary international collection of surgeons and researchers selected according to their clinical experience and expertise in thyroid surgery, parathyroid surgery, neural monitoring, and related fields. The INMSG is dedicated to improving neurophysiologic monitoring quality and preventing laryngeal nerve injury during thyroid/parathyroid surgery and provides a forum for education and dissemination of up-to-date knowledge in the field (**Table 1**, **Supplementary Files 1**, **2**). This consensus statement presents the views of the INMSG regarding the key recommendations for improving both the content and delivery of IONM surgical training and education with a goal to ensure the delivery of consistent high-quality up-to-date IONM surgical training courses from experienced leaders (**Tables 2**, **3**). We believe this consensus statement can provide societies, course directors, teaching institutions, and national organizations with a practical reference for developing IONM training programs. The consensus statement was developed under the auspices of the INMSG and is scheduled for periodic review to enable incorporation of pertinent developments in IONM knowledge and practice. Notably, the consensus statement is intended to indicate the preferred approaches to IONM training, but not necessarily the only approaches. We suggest a training course should meet the requirements listed in this consensus statement and receive approval from the INMSG Steering Committee to qualified as an “INMSG Certified Basic IONM Course” or as an “INMSG Certified Advanced IONM Course”. Considering the dynamism and the continuous evolution in the thyroid/parathyroid surgical technigues and the IONM technologies, this expert opinion provides just outlines the essential elements and key recommendations of IONM training courses intending to stimulate further discussions among all those interested in the scientific, training, clinical, and experimental aspects of IONM and to provide some guidance and clarification for ongoing discussion.

**Table 3:** Common RLN injuries models used for IONM training courses in animal laboratories.

***Traction and compression injury*** Traction stress can be experimentally induced in RLNs to observe accompanying electrophysiological EMG changes (degradation and recovery). A vascular loop (1.3-mm in width) can be wrapped around the nerve and retracted with a force gauge under varying tension force to simulate an RLN trapped against a dense, fibrous band or an RLN trapped against a crossing artery at the region of Berry’s ligament caused by medial traction of the thyroid lobe.
***Thermal injury*** During use of different energy-based devices, including activation study (activation at varying distances from the RLN) and cooling study (cooling for varying time intervals or by muscle touch maneuver before contact with the RLN), C-IONM can be used to obtain dynamic EMG data that can be used to detect thermal injury and to define the safety parameters of the tested devices.

RLN, recurrent laryngeal nerve; IONM, Intraoperative neural monitoring; C-IONM, Continuous Intraoperative neural monitoring; EMG, Electromyography.

## Conclusion

This document describes the minimum training required for teaching practical application of IONM and consensus views on key issues that must be addressed for the safe and reliable introduction of IONM in surgical practice. The intent of this publication is to provide societies, course directors, teaching institutions, and national organizations with a practical reference for developing IONM training programs. With these guidelines, IONM will be implemented optimally, to the ultimate benefit of the thyroid and parathyroid surgical patients.

## Author Contributions

C-WW, GR, MB, RS, F-YC, HD, and GD conceived and designed the study. Administrative support and provision of study materials were obtained by AKa, AKo, FF, and FW. Data collection, analysis and interpretation was done by T-YH and C-HL. All authors contributed to the article and approved the submitted version.

## Funding

This study is supported partially by Ministry of Science and Technology grant (MOST 109-2628-B-037-014), and by Kaohsiung Municipal Siaogang Hospital/Kaohsiung Medical University Research Center grants (KMHK-DK(C)110009, I-109-04, H-109-05, I-108-02), Taiwan.

## Conflict of Interest

The authors declare that the research was conducted in the absence of any commercial or financial relationships that could be construed as a potential conflict of interest.

The handling editor declared a past co-authorship with one of the authors GR.

## Supplementary Material

Supplementary File 1 INMSG IONM Training Courses information and content.

Supplementary File 2 Questionnaires for IONM Training Courses.
