# Supplementary material for: Training Courses in Laryngeal Nerve Monitoring in Thyroid and Parathyroid Surgery- The INMSG Consensus Statement
Source: Front Endocrinol (Lausanne). 2021 Jun 18;12:705346. doi: 10.3389/fendo.2021.705346 (PMC8253252; doi:10.3389/fendo.2021.705346)
Supplement: Supplementary File 1 — INMSG IONM Training Courses information and content. [file DataSheet_1.docx]

**Supplementary File 1. INMSG IONM Training Courses information and content**

| Country | Training Centers and Contact information | Training Focus |
| --- | --- | --- |
| **Germany** | **University of Duisburg-Essen, Essen**  Henning Dralle  ([henning.dralle@uk-essen.de](mailto:henning.dralle@uk-essen.de))  **University Hospital Halle, Martin-Luther-University, Haller-Wittenberg**  Rick Schneider  ([rick.schneider@uk-halle.de](mailto:rick.schneider@uk-halle.de)), | Basic IONM  Advanced IONM (intermittent; continuous) |
| U.S.A. | Massachusetts Eye and Ear Infirmary, and Massachusetts General Hospital, Harvard Medical School, Boston, Massachusetts  Gregory Randolph (gregory_randolph@meei.harvard.edu) | Basic IONM  Advanced IONM (intermittent; continuous)  Parathyroid Near-Infrared Autofloresence  Thyroid RFA |
| Poland | Jagiellonian University Medical College, Krakow  Marcin Barczyński  (marbar@mp.pl) | Basic IONM  Advanced IONM (intermittent; continuous) |
| Taiwan | Kaohsiung Medical University.  Che-Wei Wu  (cwwu@kmu.edu.tw) | Basic IONM  Advanced IONM  Experimental IONM with porcine model |
| Italy | University of Messina  Gianlorenzo Dionigi (gianlorenzo.dionigi@unime.it) | Basic IONM  Advanced IONM  IONM Simulation  IONM in Endoscopic Thyroidectomy |
