# Supplementary material for: Training Courses in Laryngeal Nerve Monitoring in Thyroid and Parathyroid Surgery- The INMSG Consensus Statement
Source: Front Endocrinol (Lausanne). 2021 Jun 18;12:705346. doi: 10.3389/fendo.2021.705346 (PMC8253252; doi:10.3389/fendo.2021.705346)
Supplement: Supplementary File 2 — Questionnaires for IONM Training Courses. [file DataSheet_2.docx]

**Supplementary File 2.** Questionnaires for IONM Training Courses

1. **Which statement regarding laryngoscopy is true?**
2. Preoperative and postoperative laryngoscopy are necessary in all cases
3. Postoperative laryngoscopy is necessary in all cases
4. Preoperative laryngoscopy is necessary in all cases
5. Preoperative and postoperative laryngoscopy are necessary only in high risk surgery
6. **According to the neuromonitoring study group, IONM can be applied to:**
7. Aid in dissection
8. Identification (neural mapping) of the RLN
9. Prognostication of postoperative neural function and lesion site identification
10. All of the above
11. **Vagal stimulation:**
12. Is needed only after dissection
13. Is less accurate than RLN stimulation for testing glottis function
14. Allows for testing the entire neural circuit
15. Can give a false-negative signal due to stimulation distal to the site of injury
16. **The optimal equipment for RLN monitoring includes:**
17. Audio and graphic monitor systems with endotracheal tube-based recording electrodes
18. Graphic monitor system and glottis observation with needle-based recording electrodes
19. Audio system and laryngeal palpation with needle-based recording electrodes
20. Audio system and glottis pressure monitoring with endotracheal tube-based recording electrodes
21. **Bipolar stimulator electrodes:**
22. Cannot be configured as dissecting instruments
23. Provide more diffuse current spread than monopolar electrodes
24. Have less sensitivity than monopolar electrodes
25. Need appropriate orientation in relation to the RLN for appropriate use
26. **During neuromonitoring the anesthesiologist should avoid:**
27. Neuromuscular blocking agents (Succinylcholine, Rocuronium, Atracurium)
28. Inhalational agents (Isoflurane, Desflurane)
29. Opioids (Remifentanil, Fentanyl, Sufentanil)
30. Propofol
31. **Which kind of neuromuscolar blocking agents are better for intubation in cases of pseudocholinesterase deficiency?**
32. Long-acting nondepolarizing muscle relaxants
33. Depolarizing muscle relaxants
34. Short-acting nondepolarizing muscle relaxants
35. Pseudocholinesterase deficiency does not affect neuromuscular blocking duration
36. **Endotracheal tube recording electrodes:**
37. Should be at the level of the subglottis
38. Belong to the stimulation side of monitoring system
39. Belong to the recording side of monitoring system
40. Are always embedded within the endotracheal tube surface
41. **Which recommendation for tube placement is true?**
42. Correct tube placement is essential to ensure optimal contact between the electrodes and vocal fold
43. The size of the endotracheal tube for neuromonitoring should be smaller than a normal endotracheal tube
44. A neuromonitoring tube cannot be placed using a stylet
45. The tube should be lubricated only with lidocaine jelly
46. **Which of these instruments has to be separated from neuromonitoring units?**
47. Ultrasound devices (e.g. Harmonic Focus)
48. Vacuum suction
49. Radiofrequency devices (e.g. Ligasure)
50. Monopolar electrocautery
51. **The endotracheal tube should be fixed**
52. After neck extension and patient positioning
53. Without neck extension
54. It should not be fixed, allowing for repositioning during surgery
55. Before neck extension and patient positioning
56. **Impedance values**
57. Are not relevant for EMG recording
58. Should be checked at the end of the dissection
59. Indicate correct endotracheal tube placement
60. Imply only good contact between the electrodes and the patient
61. **While checking monitor settings:**
62. Impedance values should be more than 5 kΩ
63. Impedance values should be less than 5 kΩ with an imbalance between electrodes of less than 1 kΩ
64. Impedance values should be less than 5 kΩ with an imbalance between electrodes of less than 5 kΩ
65. Impedance values should be less than 1 kΩ with an imbalance between electrodes of less than 1 kΩ
66. **If impedance imbalance is high, it suggests:**
67. Poor electrode-patient contact and the need for readjustment of the endotracheal tube
68. The size of the endotracheal tube is likely correct
69. Good electrode-patient contact and the possibility to start surgery
70. There is good vocal cord contact
71. **We can perform initial testing on the surgical field:**
72. With translaryngeal stimulation and/or with stimulation testing on the strap muscle
73. Just with translaryngeal stimulation
74. There are no possibilities to perform an initial testing before V1
75. Just with stimulation testing on the strap muscle
76. **Why do we test V1?**
77. To verify entire neural circuit
78. V1 is a reference for the correct placement of the endotracheal tube
79. To guarantee that paralyzing agents currently have being displaced
80. All of the above
81. **Why do we test V2?**
82. To verify entire RLN integrity
83. V2 is a reference for the troubleshooting algorithm
84. To discriminate between type 1 and type 2 lesions
85. All of the above
86. **Which is the correct sequence for IONM?**
87. L1 – V1 – R1 – R2 – V2 – L2
88. V1 – R1 – R2 – V2
89. L1 – R1 – V1 – V2 – R2 – L2
90. L1 – L2 – V1 – V2 – R1 – R2
91. **If the RLN is being stimulated and EMG activity is not present or lower than 100μV, it is necessary to:**
92. Stimulate the ipsilateral vagus nerve to look for a twitch response. If it is not present, the problem is in the recording side
93. Stimulate the ipsilateral vagus nerve to look for a twitch response. If it is present, the problem is in the recording side
94. Stimulate the ipsilateral vagus nerve to look for a twitch response. If it is present, the problem is in the stimulation side
95. Stimulate the contralateral RLN to look for a twitch response. If it is present, the problem is in the recording side
96. **If ipsilateral RLN stimulation is not giving adequate EMG signal but there is twitch response, then during stimulation of the contralateral vagus nerve:**
97. Poor EMG signal likely means a recording side problem
98. Good EMG signal likely means a recording side problem
99. Stimulating at greater than 10 mA could be useful
100. It is not important to achieve contralateral vagus nerve signal, even in bilateral procedures
101. **If laryngeal twitch is absent:**
102. We should consider a recording side error
103. We should consider a stimulation side error
104. For sure it is not due to a non-recurrent laryngeal nerve
105. The RLN is surely damaged
106. **We can suspect LOS if there is no EMG response or if the EMG amplitude is**
107. < 10 μV
108. < 500 μV
109. < 1000 μV
110. < 100 μV
111. **LOS is defined as**
112. No laryngeal twitch and/or observed glottis twitch
113. EMG change from initial satisfactory EMG
114. No or low response (i.e., 100 μV or less) with stimulation at 1 mA in a dry field
115. All of the above
116. **Correct RLN stimulation is achieved with an intensity of**
117. 0.3 mA
118. 1 mA
119. 10 mA
120. 30 mA
121. **LOS can be interpreted**
122. Only if laryngeal twitch is present
123. Only if you achieve a good EMG value initially
124. Even though initial EMG is not achieved
125. Only if the EMG waveform is lost
126. **The INMSG suggests an initial value for V1**
127. 500 μV
128. < 500 μV
129. < 100 μV
130. 100 – 300 μV
131. **In the case of LOS, if we do not obtain a good signal on the contralateral vagus nerve:**
132. It is always necessary to perform a staged thyroidectomy
133. It is probably a problem of endotracheal tube position
134. It is probably a bilateral injury
135. It is a true LOS
136. **In the case of LOS, we have to map the nerve to look for the lesion. Which of the following is true?**
137. A type 3 lesion is a global injury of both the recurrent and vagus nerves
138. A type 2 lesion is always due to thermal injury
139. A type 1 lesion stands for a global injury, and a type 2 lesion stands for a segmental injury
140. A type 1 lesion stands for a segmental injury, and a type 2 lesion stands for a global injury
141. **Which of the following is a stimulation error?**
142. Transtracheal stimulation
143. Probe malfunction
144. Insufficient stimulator current
145. All of the above
146. **Which of the following regarding amplitude is true?**
147. It varies significantly among different patients
148. It varies only in cases of nerve damage
149. It is always stable during intraoperative neuromonitoring
150. It varies only with increasing intensity of the stimulation (mA)
151. **Minimal threshold is defined as:**
152. The current that, applied to the nerve, robustly triggers an EMG signal
153. The current that, applied to the nerve, first starts to trigger minimal EMG activity
154. The current that, applied to the nerve, first starts to trigger physiologic EMG activity
155. The current that, applied to the nerve, first starts to trigger maximal EMG activity
156. **Latency:**
157. Represents the number of the fibers participating in the depolarization event
158. Depends on the distance of the stimulation point to the contralateral vocal cord
159. Does not depend on the distance of the stimulation point to the ipsilateral vocal cord
160. Represents the speed or ease of stimulation-induced depolarization
161. **Which of the following is not a cause for a false positive result?**
162. Blood or fascia covering the stimulated nerve segment
163. Endotracheal tube displacement
164. Vocal cord paralysis with early neural recovery
165. Distal stimulation relative to the injured nerve segment
166. **Which of the following is a cause for a false negative result?**
167. Injury subsequent to last testing stimulation or delayed neuropraxia
168. Endotracheal tube displacement
169. Low stimulation current
170. Paralytic agent
171. **With regards to EBSLN stimulation with the use of a standard nerve-monitoring endotracheal tube, the following is true regarding the presence of a CTM twitch and glottic waveform:**
172. A visible CTM twitch can be identified in 100% of cases, and EMG activity can be quantified in nearly 80% of cases
173. A visible CTM twitch can be identified in 80% of cases, and EMG activity can be quantified in nearly 100% of cases
174. A CTM twitch is always identified, but EMG activity is rarely quantified
175. EMG activity is always identified, but a CTM twitch is rarely observed
176. **With stimulation of the EBSLN, the mean amplitude of the EMG response recorded on endotracheal tube surface electrodes is:**
177. About 1/3 of the RLN amplitude
178. About twice the RLN amplitude
179. About 1/2 of the RLN amplitude
180. Unable to be measured
181. **According to the INMSG, the nerve stimulation pattern that maximally assures EBSLN preservation includes:**
182. Clearly present stimulation of the EBSLN cranially and medial to the evolving superior pole pedicle
183. Clearly absent stimulation of the EBSLN within the superior pole pedicle that is to be divided
184. Both the first and second answer choices
185. None of the above

**Answers: (1-10): a d c a d a c c a d**

**(11-20): a d b a a d d a b b**

**(21-30): b d d b b a b d d a**

**(31-37): b d d b a a c**
